# Supplementary material for: Complex‐centric proteome profiling by SEC‐SWATH‐MS
Source: Mol Syst Biol. 2019 Jan 14;15(1):e8438. doi: 10.15252/msb.20188438 (PMC6346213; doi:10.15252/msb.20188438)
Supplement: Supplementary file 8 — Dataset EV7 [file MSB-15-e8438-s008.zip › feature_plots_string/Q6W5P4.pdf]

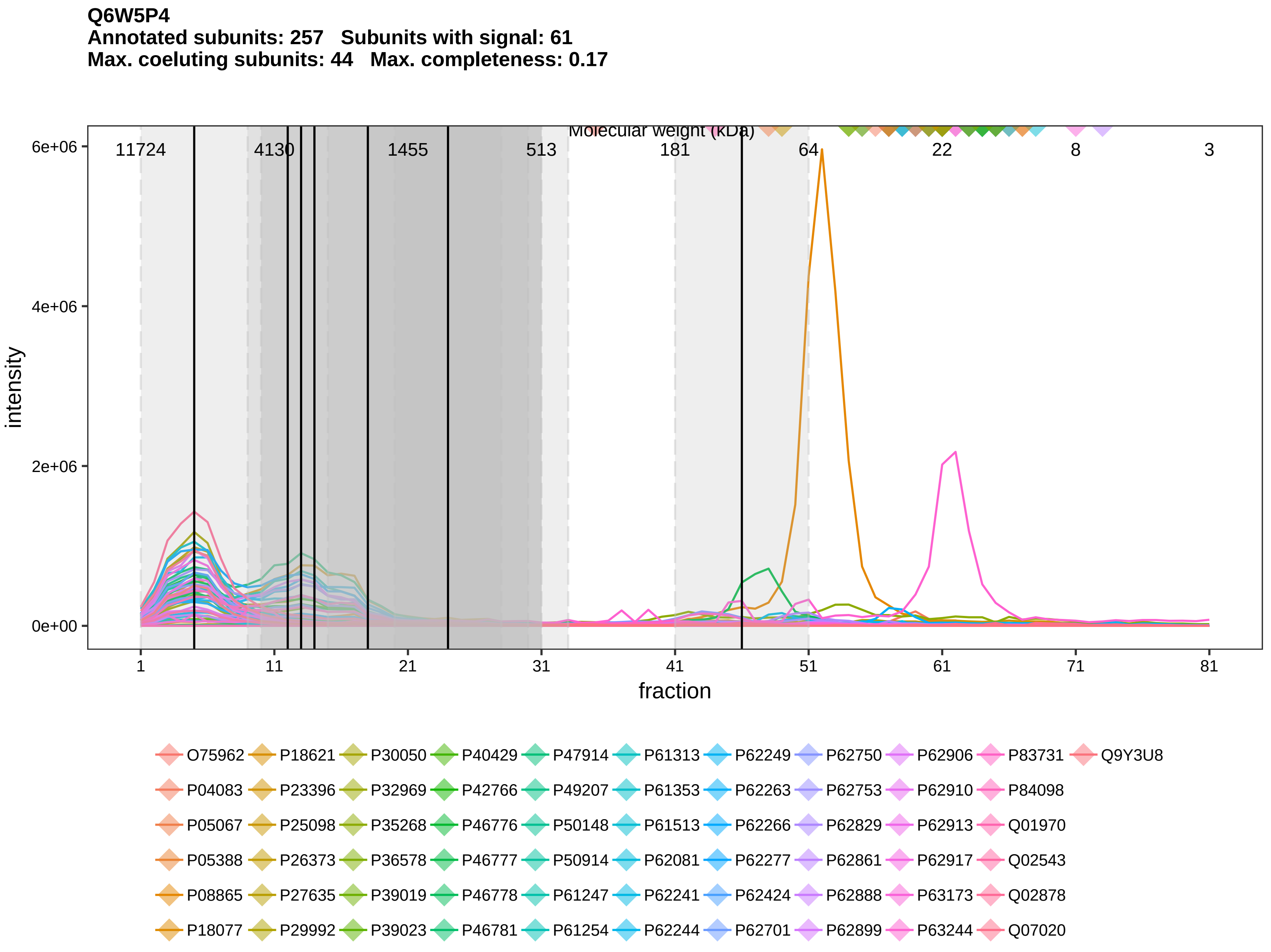

Q6W5P4
Annotated subunits: 257 Subunits with signal: 61
Max. coeluting subunits: 44 Max. completeness: 0.17
Molecular weight (kDa)
11724
4130
1455
513
181
64
22
8
3
intensity
6e+06
4e+06
2e+06
0e+00
fraction
1
11
21
31
41
51
61
71
81
O75962
P04083
P05067
P05388
P08865
P18077
P18621
P23396
P25098
P26373
P27635
P29992
P30050
P32969
P35268
P36578
P39019
P39023
P40429
P42766
P46776
P46777
P46778
P46781
P47914
P49207
P50148
P50914
P61247
P61254
P61313
P61353
P61513
P62081
P62241
P62244
P62249
P62263
P62266
P62277
P62424
P62701
P62750
P62753
P62829
P62861
P62888
P62899
P62906
P62910
P62913
P62917
P63173
P63244
P83731
P84098
Q01970
Q02543
Q02878
Q07020
Q9Y3U8
